# Evaluating the re-identification risk of a clinical study report anonymized under EMA Policy 0070 and Health Canada Regulations

**DOI:** 10.1186/s13063-020-4120-y

**Published:** 2020-02-18

**Authors:** Janice Branson, Nathan Good, Jung-Wei Chen, Will Monge, Christian Probst, Khaled El Emam

**Affiliations:** 1 Novartis, Basel, Switzerland; 2 Good Research, El Cerrito, CA USA; 3 Privacy Analytics, Ottawa, Canada; 4 Children’s Hospital of Eastern Ontario Research Institute, Ottawa, Canada

## Abstract

**Background:**

Regulatory agencies, such as the European Medicines Agency and Health Canada, are requiring the public sharing of clinical trial reports that are used to make drug approval decisions. Both agencies have provided guidance for the quantitative anonymization of these clinical reports before they are shared. There is limited empirical information on the effectiveness of this approach in protecting patient privacy for clinical trial data.

**Methods:**

In this paper we empirically test the hypothesis that when these guidelines are implemented in practice, they provide adequate privacy protection to patients. An anonymized clinical study report for a trial on a non-steroidal anti-inflammatory drug that is sold as a prescription eye drop was subjected to re-identification. The target was 500 patients in the USA. Only suspected matches to real identities were reported.

**Results:**

Six suspected matches with low confidence scores were identified. Each suspected match took 24.2 h of effort. Social media and death records provided the most useful information for getting the suspected matches.

**Conclusions:**

These results suggest that the anonymization guidance from these agencies can provide adequate privacy protection for patients, and the modes of attack can inform further refinements of the methodologies they recommend in their guidance for manufacturers.

## Introduction

There is growing recognition within the research community that the re-analysis of clinical trial data can provide new insights compared to the original publications [1]. Evidence from voluntary data-sharing efforts that have been running over the last few years suggest that the validation of the primary endpoint is an uncommon objective of secondary analysis of clinical trial data, and that the most common purposes for secondary analyses are new analyses of the treatment effect and the disease state [2].

Clinical trial data mean two different things. First, there are the structured individual-level participant data and, second, there are the clinical reports. Clinical reports would normally follow ICH guidance M4 for Clinical Technical Documents [3], and module 5 of these documents is the clinical study report (CSR), which would normally follow ICH guidance E3 [4].

Regulators at the European Medicines Agency (EMA) have issued Policy 0070 requiring the release of clinical reports [5]. When a manufacturer applies for a centralized marketing authorization at the EMA, the Committee for Medicinal Products for Human Use (CHMP) provides the (positive or negative) recommendation to the European Commission (EC). The EC grants or refuses the marketing authorization in a centralized procedure. The anonymized clinical reports are then published online after the EC decision,^1^ or after the CHMP decision if there is no EC decision. A future phase of Policy 0070 is expected to address the release of individual participant data, but at the time of writing no date has been set for this.

Similarly, Health Canada’s Public Release of Clinical Information (PRCI) initiative [6] that went into effect in 2019 requires the release of anonymized clinical reports after a final regulatory decision is made. However, it also includes requests from the public for legacy clinical reports within its scope. The anonymized documents are published on the Health Canada portal.^2^

The secondary analysis of clinical reports has produced informative research results, including those on drug safety, evaluating bias, replication of studies, and meta-analysis [7].

Anonymization is necessary before clinical reports are released by these agencies on their portals because they can contain a substantial amount of personal health information. For example, there will be detailed information about participant medical histories, and narratives documenting adverse events as well as any relevant information needed to interpret these adverse events. These documents also contain summary tabular data (e.g., vital statistics and counts). It is known that personal information can be derived from tabular data and there is a body of work on assessing the re-identification risk and the anonymization of tabular data [8–10].

Appropriate anonymization protects participant privacy, and also limits the liability of the manufacturer and the regulatory agencies when this information is made broadly available. Furthermore, using information that has been released publicly but that may not have been anonymized adequately by the sponsor can also impose legal risks on the users of that information [11], such as researchers and journalists.

The EMA has published guidance for manufacturers to anonymize their documents before submitting them to the Agency under Policy 0070 [12]. Health Canada’s anonymization guidance follows the same principles as the EMA’s [6], and they will accept documents already anonymized according to the EMA’s recommended methodology.

In this context, anonymization means ensuring that the probability of correctly assigning an identity to a participant described in the clinical reports is very small. This is also referred to as the probability of re-identification. Both the EMA and Health Canada have set an acceptable probability threshold at 0.09.

The EMA anonymization guidance recommends a risk-based approach to anonymization, and allows for two approaches: a quantitative approach and a qualitative approach. The former entails using statistical disclosure control techniques to estimate the actual probability of re-identification (e.g., see [8–10, 13–16]). A qualitative approach as has been applied in practice does not estimate probabilities, but uses qualifiers as low/medium/high risk. The risk level is determined using criteria such as the number of participants, whether the trial is in a rare disease, subjective assessment of potential socioeconomic harm to patients if there is re-identification, and the perceived re-identification risk of certain pieces of information (whether they would be knowable by potential adversaries).

To date, 61% of dossiers published under Policy 0070 have followed a qualitative approach and 10% a quantitative approach [17] (the remainder did not require anonymization, such as systematic reviews). There is no generally accepted methodology for qualitative anonymization of clinical trial information and therefore each of the manufacturers that has published dossiers on the EMA portal using a qualitative approach has developed their own methodology. In addition to questions about the validity of custom home-grown methodologies, there is the practical challenge of pooling information across trials if they are anonymized differently. On the other hand, there is a large body of literature on quantitative anonymization. Health Canada is emphasizing the need for quantitative methods for re-identification risk measurement and anonymization [6].

One concern that has arguably been contributing to the slow adoption by manufacturers of quantitative anonymization approaches described in the EMA and Health Canada guidance is uncertainty on whether they are sufficiently privacy protective [18]. The purpose of the current study was therefore to empirically test whether the quantitative anonymization approach for CSRs described in the EMA and Health Canada guidance is sufficiently privacy protective [18]. This study makes two contributions: it is the first empirical evaluation of re-identification risk for a CSR, and it is the first empirical test of the hypothesis that the EMA and Health Canada anonymization guidance provides adequate privacy protection.

Our empirical evaluation of re-identification risk follows a UK methodology described by the Information Commissioner’s Office (ICO) [19], the Office of National Statistics (ONS) [20], and the UK Anonymisation Network [21]. Furthermore, in the context of deciding whether information is personal, a tribunal judge recently used the success of such an empirical re-identification evaluation as the primary criterion [22]. Our study is consistent with previous empirical re-identification risk evaluation studies in that it focuses on data subjects in a single dataset [23–35]. In Additional file 1, we review previous work in this area.

This paper is structured as follows. We first describe the specific trial that was the target of the empirical test and the methods that were used to re-identify data subjects, including the metrics collected about the success rate and effort. This is followed by the results, limitations, and conclusions.

## Methods

### Study design

The basic design of this study involves taking an anonymized CSR (following the approaches described by the EMA and Health Canada) and then subjecting it to re-identification by attempting to match the participants in the CSR with individuals in the real world. The group performing the anonymization was independent from the group performing the re-identification. This section describes the CSR, the matching, and how it was evaluated.

### The clinical study report

The CSR that was the subject of the empirical re-identification test pertained to a clinical trial of nepafenac. Nepafenac is a non-steroidal anti-inflammatory drug that is sold as a prescription eye drop under two main trade names, which differ on the basis of drug concentration—the 0.3% suspension is marketed as Ilevro, while a 0.1% suspension is marketed as Nevanac. Nevanac received FDA approval in August 2005, while Ilevro was approved in October 2012.

The trial in question (C-12-067) was a randomized, double-masked, controlled study to assess the safety and efficacy of the nepafenac ophthalmic suspension (0.3%) for improvements in clinical outcomes among diabetic subjects following cataract surgery. The trial was sponsored by Alcon Research, Ltd, currently a division of Novartis Europharm Ltd.

The trial ran from 26 March 2013 to 13 May 2015 in 66 centers in the USA, Latin America, and the Caribbean. Subjects must have been 18 years of age or older, must have had a cataract, and must have been planning to undergo cataract extraction by phacoemulsification. Subjects must also have had a history of diabetes and diabetic retinopathy. There were 615 subjects randomized and 598 were included in the primary efficacy analysis. The distribution of subjects by country is presented in Table 1.

**Table 1:** Distribution by country of nepafenac trial participants

Country	Number of subjects	% of total subjects
United States	500	83.6
Panama	26	4.3
Puerto Rico	54	9
Other	18	3
Total	598	100

This trial was selected because it has a large number of participants in the USA. Most re-identification studies have been performed on US data subjects [23], arguably because there are more data available about them to make such attempts more likely to succeed. Furthermore, an anonymized version of this particular CSR had been submitted to the EMA under their Policy 0070. This meant that the anonymization team had experience working with it and were able to update the anonymization applied for the current study using recent methodological advances (e.g., by using active learning methods to improve information extraction for the detection of personally identifying information [36]).

### Anonymization of the CSR

The CSR was anonymized following the EMA Policy 0070 guidelines [12], and the anonymized document was made available for our study. The anonymization performed is also consistent with the Health Canada PRCI guidelines [6]. The anonymization was performed by a team from Privacy Analytics Inc. in Canada. The general quantitative anonymization methodology has been described in detail elsewhere [13, 37–40].

Specifically, a hybrid approach for information extraction consisting of a rule-based engine [41] and an active learning system [36] was used to extract subject identifiers and quasi-identifiers (e.g., dates, participant demographics, medical history, and serious adverse events) from the CSRs. All subject identifiers were pseudonymized. A sample of pages were also manually annotated by two independent annotators—evidence shows that the accuracy diminishes with more than two identifiers [42]. The manual annotations were used to create a gold standard from which recall (the proportion of identifiers detected correctly) was computed. The probability of re-identification was measured using a *k*-anonymity estimator [43]. A risk model for unstructured text was then used to estimate the overall risk of re-identification taking into account the recall and the *k*-anonymity results [44]. If the upper 95% confidence limit of the estimated risk was larger than the EMA and Health Canada recommended threshold of 0.09, then transformations were performed on the quasi-identifiers until the estimated upper risk limit was at or below 0.09. The transformations performed were generalization and suppression.

### Suspected matches vs. verified matches

During a re-identification attempt, there is first a suspected match with a real identity which would then need to be verified to ensure that it is a correct match. An example of a verification in the current study could be if the pharmaceutical company (manufacturer) had the correct identity of the patient and was able to confirm whether a suspected match was correct. Counting only the suspected matches will give quite conservative results, in that the counts will overestimate the match rate. In practice, there is a sharp drop in the match rate between the suspected results and the verified results. The summary presented in Table 2 provides the success rates of verifications from previous studies.

**Table 2:** Rate of correct verification from suspected matches

Study	Data details	% of suspected matches verified as actual matches
Kwok and Lafky and colleagues [25, 26]	Matched 15,000 Safe Harbor de-identified admission records from a regional hospital to a marketing dataset of 30,000 records	10% (2/20)
Elliot et al. [29]	Sampled records from the UK Labour Force Survey (LFS) and the Living Costs and Food Survey (LCF) to re-identify. Matches were performed with and without the Output Area Classifier (OAC), which provides more precise geography	• LFS: 12% (6/50) using web-based info to match with;28% (14/50) using commercial data • LCF: 10% (2/20) for dataset without OAC;43% (18/42) for dataset with OAC
Tudor and colleagues [30, 31]	Data examined were tabular in nature, consisting of 89 tables that were determined to be potentially high risk	• 36% claims of identifying a neighbor were correct • 61% correct for identifying self/family • All claims, except one, involved people the intruder knew
Sweeney [45]	News reports of hospitalizations (*n* = 81) were used to identify individuals in a Washington state hospital inpatient dataset of 648,384 records	23% (8/35)

In the context of clinical trials, manufacturers only get key coded data from the trial sites and not names and addresses of participants. Because the manufacturer does not know the identity of the participants, it is not possible for the manufacturer to directly verify whether a suspected match is correct or not.

Under such conditions there are three approaches that can be used to obtain or estimate a count of the verified matches:

1. The manufacturer can go through each individual site and have them verify each suspected match many years after the trial has been completed.
2. Assign a confidence score to assess the likelihood that the suspected match was correct. A commonly used confidence scale is a number from 1 to 5, with 5 indicating high confidence in the match as illustrated in Table 3 [30]. The confidence percentages and qualitative meaning were based on the actual subjective scores and terms used by analysts performing re-identification in previous studies. Therefore, they are grounded in the manner in which analysts express themselves with respect to suspected matches. In the table we also interpret the meaning of each of the five levels into low/medium/high confidence in a suspected match. The confidence score has been found to be correlated with the correctness of the match after verification [30].
3. Using the rates from literature presented in Table 2, compute the weighted mean of suspected matches that are verified matches and use that as an adjustment. This value is 23% (i.e., 23% of suspected matches are verified). Although it is not clear whether the suspected matches here were only the high confidence ones (i.e., verifications in these studies were only attempted on high confidence suspected matches), making this at best another ceiling estimate.

**Table 3:** Interpretation of the confidence levels attached to candidate matches [30]

Confidence level	Confidence percentage	Meaning in words	Interpretation
1	0–19	Not at all confident, complete guess	Low confidence
2	20–39	Not very confident, bit of a guess	
3	40–59	Not quite sure, uncertain	Medium confidence
4	60–79	Fairly sure, reasonably confident	High confidence
5	80–100	Very confident, absolutely sure	

For the current study we used the second approach (confidence scores).

### Third party

The re-identification study was performed by an independent third party who was not involved in any way in the anonymization of the data itself, namely a team from Good Research in the USA.

The third party performing the re-identification did not convey the suspected re-identifications back to the study sponsor nor to the group that performed the anonymization. Only the quantitative and summary results were communicated back—the same results as presented in this paper.

### Target subjects

The re-identification study was performed only on the 500 participants based in the USA. There are three reasons for using the US patients as the target participants:

1. As noted earlier, most known re-identification attacks have been performed in the USA (see [23]) because there is more public information available about the population, making such attacks easier. Arguably, then, the results from US participants would represent the ceiling success rate.
2. For practical reasons, the study needed to be performed on patients living in an English-speaking country.
3. The largest geography in this trial was the USA, providing a larger sample of target individuals.

### Methods used

The terminology used in the UK to describe the commissioned re-identification attack on a dataset is a *motivated intruder test* [19, 20]. We will also use that terminology here to be consistent with the literature.

The ICO guidance notes that the motivated intruder should not have specialized knowledge [19]. However, there was a minimum amount of domain knowledge that our investigators needed to have to proceed with the study, for example, where to start to look for public facing clinical information and how and what kinds of Freedom of Information requests to attempt.

During risk measurement and anonymization there are two directions for a re-identification attack that need to be considered [13]. An attack can use information from an external source and match that with the information in the CSR (population-to-sample attack). In the context of a motivated intruder test, this could be a famous person or an acquaintance of the intruder. An attack can also start from the characteristics of a patient in the CSR and attempt to match it with person profiles in the external data sources or registries (sample-to-population attack). The registry may be pre-existing or may be created by conducting searches on the Web. Commercial databases would also be considered a kind of registry.

We defined several approaches to re-identify individuals in the dataset. In practice, the process was iterative, where partial information of any person known to have participated in the clinical study was gathered from multiple sources. The partial information was then combined to attempt to re-identify the individuals. Also note that these approaches were informed by discussions within the clinical trial disclosure community with respect to methods and sources that were believed to be useful for actual re-identification attacks.

The following were the approaches that were examined by the analysts performing the re-identification attempt:

1. Clinical reports: identifying external clinical reports of adverse events in registries and released by regulatory agencies, matching external reports with more information to the anonymized events in the CSR.
2. FDA and EMA Freedom of Information Act (FOIA) requests: requesting records from federal (US) and EU agencies.
3. Death records: given that there were five deaths among the subjects, matching these to public death records could provide additional identity information.
4. Hospital discharge records: by identifying some of the areas where the study was performed, we may obtain matches from these to the adverse events on the anonymized report.
5. Re-contacting subjects: attempting to recruit the subjects from the same study again.
6. Social media: although the subjects may have been told to not post information from the studies, some individuals may have posted information directly or indirectly related to it, leading us to partially identify some subjects
7. Voter registration records: as outlined by Benitez and Malin [46], voter registration records can provide a possible avenue for re-identifying medical records at scale.
8. Other approaches.

Additional file 2 details the goals, external datasets used, and methods of attack for each of these approaches. According to the ONS guidance, the intruder should spend a few hours to re-identify a record [20].

### The outcome and its interpretation

At the end of a motivated intruder test there are two summary numbers that need to be generated:

1. the percentage of individuals in the dataset that have a suspected match
2. the effort to find a suspected match

Each of these will be described further below.

#### Percentage of individuals with a suspected match

The denominator for this calculation is 500. We did not consider incorrect re-identification in the final calculation. Although in theory incorrect re-identification can cause the data subjects some harm, there is no way to really protect against incorrect re-identification short of not sharing any data. An adversary can assign random names to records in a database and end up with many incorrect re-identifications. Therefore, we focus only on correct (suspected) re-identification.

#### Effort to re-identify an individual

With respect to the effort to re-identify an individual, this was calculated as an average across all individuals who were candidates to be re-identified. In this case, the total effort would be the attempted effort for the failed matches as well.

## Results

### Suspected matches

Six subjects were determined to be suspected match candidates, with confidence scores all within the “low confidence” group. The successful approaches are summarized in Tables 4 and 5. As can be seen, only the search through death records and social media searches identified suspected matches.

**Table 4:** Approaches used for each of the six suspected matches

Approach	External source	Confidence score	Confidence group	Reason for confidence score
Social media	Facebook	2	Low	Date of surgery + location + symptoms + diabetic (inferred)
Social media	Reddit	1	Low	Date of surgery + age + gender + diabetic
Death records	Ancestry.com	1	Low	Age + date of death + ethnicity (inferred) + unknown diabetic status
Death records	Ancestry.com	1	Low	Age + date of death + ethnicity (inferred) + unknown diabetic status
Death records	Ancestry.com	1	Low	Age + date of death + ethnicity (inferred) + unknown diabetic status
Death records	Ancestry.com	2	Low	Age + diabetic (inferred) + details of death + location

**Table 5:** Summary of re-identification confidence scores

Confidence score	Count	% of total subjects
1	4	0.8
2	2	0.4
3	0	0
4	0	0
5	0	0
Partially verified	0	0
Fully verified	0	0

Using the approach of matching against death records, four potential matches were obtained, three of which had a confidence score of 1 and the other a confidence score of 2. The confidence score was determined by expert assessment based on the fit of the match, such as the obituaries and narratives with the records on the anonymized report or other known information based on the study (e.g., being diabetic and having had cataract surgery), age, gender, and cause of death.

Most of the initial hits for Facebook and Reddit keyword searches were discarded due to their low probability of being in the study (not known to be diabetic) and the lack of specific identifiers to single them out in the CSR. The search keywords used are detailed in Additional file 4. However, it was possible to identify two subjects for which there was some confidence of a match based on use of the drug, surgery, date of surgery, suspected or confirmed diabetic condition, and additional information from other clinical/medical visits, although the confidence scores were only 1 and 2.

The ONS guidance [20] states that “[t]he aim is not to release a dataset with zero risk so a good result would be if there were a small number of [re-identifications] with low confidence.” Such a result indicates that the re-identification is low and that the perturbations applied to the anonymized data were not too extensive. Therefore, the results obtained here are consistent with this balance.

Given that all of the suspected matches had a low confidence score, the likelihood of an attempted verification would be low.

### Re-identification effort

A total of 170 h was spent on the investigation and the subsequent report (this does not include the effort spent writing the current article). Approaches #3 (death records) and #6 (social media) resulted in potential matches, and overall took 49 and 75 h, respectively. Details of the time breakdown and process are described in Additional file 3.

The total estimated effort per subject was approximately 24 h. This was calculated by aggregating the entire effort (170 h) excluding 25 h of project management tasks (e.g., writing the report, project meetings) for a total of 145 h. This number is divided by the six candidates: 145 h / 6 candidates = 24.2 h per candidate.

No commercial datasets were purchased nor were any other expenditures incurred for the purpose of the re-identification, therefore no additional costs beyond labor costs were reported.

## Discussion

### Summary

There has been good progress recently in making the reports from clinical trials publicly available through the EMA and Health Canada. But there have also been concurrent concerns about the protection of participant privacy. Medical histories and narratives in clinical study reports can include very sensitive and detailed information about trial participants. While clinical trial participants are supportive of data sharing as long as adequate safeguards are in place [47], for specific diseases and conditions, patients worry about discrimination in employment, reduced access to insurance including health care, and inability to secure loan and credit advances if their sensitive information is identified [48]. In general, it is known that when patients have concerns about the privacy of their personal health information, they adopt privacy protective behaviors, such as not seeking care, hiding information, and visiting multiple providers [49]. If patients worry about how their data are used, there is the risk that this will affect their willingness to participate in trials. The approach that has been adopted to mitigate this risk and enable access to clinical reports is anonymization.

The purpose of this study was to empirically test the hypothesis that a clinical study report that was anonymized according to the quantitative methods described by the EMA and Health Canada for the public release of documents provided adequate privacy protection to participants. The drug in question was nepafenac, which is a non-steroidal anti-inflammatory drug that is sold as a prescription eye drop. The commissioned re-identification focused on the 500 patients who were recruited in the USA, and was performed by an independent third party not involved in the anonymization of the documents and that had no vested interest in the outcome.

Overall, there were six suspected re-identifications with low confidence scores. The scoring scheme has been correlated with the correctness of suspected re-identification in previous work. The interpretation of this result is that no patients could reasonably be re-identified using the re-identification methods described here.

This attack provides confidence that the quantitative anonymization approaches outlined in the EMA and Health Canada guidance can be a reasonable approach to protect patient privacy. However, it should be noted that many sponsors do not use a quantitative approach to anonymize their submissions to the EMA and therefore our conclusions on managing privacy risks do not extend to the case where qualitative or other approaches are used. The impact of this anonymization approach on the utility of the anonymized documents is not known as that was not the subject of this study, but should be examined in future research.

### Limitations

An empirical re-identification risk assessment has its own limitations in that it cannot mimic exactly what an adversary will do when attacking a dataset. For example, an adversary with criminal intent may do things that we would not in a commissioned re-identification attempt, for example, committing criminal acts or buying stolen data to match against the CSR. Also, every commissioned attack has a budget and time limitations, and that imposes some boundaries on what can be achieved. It is plausible that an adversary will have more budget than that assumed in the current study. Therefore, there are legal, ethical, and practical limits to what can be achieved using such an empirical test.

This study was performed on subjects in the USA. Different results may have been obtained had the motivated intruder test been on subjects from a different country. Although, arguably, the match rates would be lower in other countries and therefore our numbers should be considered a ceiling.

It is not known at this point whether a motivated intruder test on subjects in a rare disease trial or a different therapeutic area would produce similar results. Therefore, we are cautious in generalizing the findings across therapeutic areas and studies investigating small and narrower populations. Furthermore, our analysis was performed on a single clinical trial and a single CSR. Caution should be exercised when generalizing these findings more broadly to other trials and CSRs. Our study nevertheless provides some initial evidence as well as a re-identification methodology specific to clinical trials that can be applied in future work.

The EMA and Health Canada guidance documents do not provide operational step-by-step directions on how to perform anonymization—they generally refer to the literature for these details. To the extent that the interpretations of the agency guidelines are heterogeneous, other similar studies may achieve different success rates with their re-identification attempts. In particular, the team that performed the anonymization on this CSR were quite knowledgeable of the field and methods of anonymization. Since no minimal expertise requirements are stipulated by the regulatory authorities, other teams performing the anonymization, even if following the same guidance, may achieve different results.

## Conclusions

This study was the first to empirically test the anonymization methods that have been recommended by the EMA and Health Canada to facilitate the sharing of clinical reports more broadly. The results are encouraging in that they demonstrate the robustness of these anonymization methods. Additional empirical tests of re-identification risk on anonymized CSRs will accumulate evidence on the strengths and weaknesses of the quantitative approach to anonymization that is described by the two agencies.

It is also generally recommended that manufacturers regularly perform re-identification studies on their documents and data, especially when they release them in the public domain. Such empirical feedback will help improve the anonymization methods that are used, and can augment the statistical risk estimation models that are typically used to determine the level of perturbation and redaction that needs to be applied.

## Supplementary information

**Additional file 1.** Review of commissioned re-identification attacks.

**Additional file 2.** Attack methods used.

**Additional file 3.** Effort diagram per approach.

**Additional file 4.** Search keywords.

## Data Availability

A version of the nepafenac anonymized CSR is available on the EMA portal (registration required): https://clinicaldata.ema.europa.eu/web/cdp/home

## Footnotes

1 See: https://clinicaldata.ema.europa.eu/web/cdp/home

2 See https://clinical-information.canada.ca/search/ci-rc
