# Supplementary material for: Evaluating the re-identification risk of a clinical study report anonymized under EMA Policy 0070 and Health Canada Regulations
Source: Trials. 2020 Feb 18;21:200. doi: 10.1186/s13063-020-4120-y (PMC7029478; doi:10.1186/s13063-020-4120-y)
Supplement: Supplementary file 1 — Additional file 1. Review of commissioned re-identification attacks. [file 13063_2020_4120_MOESM1_ESM.pdf]

# Additional file 1: Review of Commissioned Re-identification Attacks

In this appendix we review the literature on commissioned re-identification attacks on different types of data. While the “motivated intruder” terminology has not been consistently used in this literature, the examples below can all reasonably be characterized as such. Note that this review excludes attacks on datasets that were not de-identified according to a known standard or guideline.[1]

El Emam, et al., reviewed successful re-identification attacks up to 2011, which included 14 published attacks, with six of them on health data [2]. They found that the success rate for all re-identification attacks included in the study was approximately 26%, and 34% for health data specifically; however, when examining the studies more closely, it was found that only 2 of the 14 attacks were performed on data that were de-identified according to current standards while the other 12 studies targeted pseudonymized data. In both of the two attacks on de-identified data, the risk of re-identification was found to be very low. In the first case, the authors matched sample records from the UK Census with records from the general household survey [3]. The authors of the study verified their matches through the Office of National Statistics (ONS) which was privy to the corresponding individuals' identities. They found 219 unique matches between the datasets, 112 with 2 possible matches, and were able to verify 8 correct matches. It was not clear from this study what the exact proportion of records that could be re-identified might be but as we have seen, the number of matched records was quite small.

The second case was commissioned by the Department of Health and Human Services in the US to determine the re-identification risk of data de-identified using the HIPAA Safe Harbor standard [4], [5]. The researchers used a patient data set that consisted of 15,000 Safe Harbor de-identified admission records from a regional hospital and matched these against a marketing data set of 30,000 records with similar attributes purchased from InfoUSA, a marketing research firm. All of the records were from individuals who self-identified as Hispanic and the variables that were used to match records included age or year of birth, sex, the first 3 digits of ZIP codes, and marital status. The best results were obtained when matching on age (vs. year of birth) and other attributes. They found 20 patient records that matched to 22 records in the marketing data set, with 2 of these confirmed to be “true” identity matches. That is equivalent to a re-identification rate of 0.013% [4].

Elliot undertook a similar study in 2007, linking the Sample of Anonymised Records (SARs) from the 2001 UK census to the microdata from the Spring 2001 UK Labour Force Survey (LFS) [6]. The focus of this test was to assess whether the statistical disclosure control (SDC) methods used on the 2001 SARs protected against the risk of re-identification posed by linkage with external datasets. All unique matches found between the datasets were sent to ONS for verification. Between the released SARS file and the LFS, 3130 matches were found; however, no name or address could be found for many of these matches, reducing the number that could be verified to 2234. Of these matches, 51 were verified to be correct re-identifications (2.28%). When using a fishing method of attack to target high risk records in the

[1] For example, in one study on public files from the Personal Genome Project (PGP) found a large number of the files with embedded names of individuals [1]. Therefore these files were clearly not de-identified.

data, Elliot had a similarly low level of success in re-identifying individuals. Elliot concludes that “the SDC that was employed on the 2001 SARs appears to seriously undermine intrusion attempts” [6].

In a 2011 study, a disclosure risk analysis of the supporting people dataset disseminated by the Department for Communities and Local Government (DCLG) was undertaken by Elliot [7]. “Cross matching attacks” and “Response knowledge based attacks” were examined in his analysis [7]. Similar to the previous study, the cross matching attack used a large data set of identifiable information to match and re-identify records in a de-identified data set. For this type of attack, Elliot aimed to uncover “the probability of a correct match given a unique match” [7]. In other words, what is the probability of a cross match being a “true” match that actually identifies an individual in the data set? For this data set, he found that the probability was very low: 0.0177. However, the response knowledge based attack increased the risk considerably due to the high number of unique records in the data set (12.18%). In this type of attack, an intruder would know that a particular individual is included in the data set. Therefore, if the individual is unique in the data set then they could be easily identified by an intruder.

In another study [8], Elliot and his colleagues simulated an attack by an intruder who has response knowledge about an individual in the data set, focusing on samples from two social service databases: the UK Labour Force Survey (LFS) and the Living Costs and Food Survey (LCF). They used web-based information and a commercial database to re-identify 50 sampled records from each survey. For the LFS, they found that they were able to correctly match 6 of the 50 sampled records (12%) using web-based information alone and 14 (28%) when the commercial data was used as well [8]. The LCF included an Output Area Classifier (OAC) which was not included in the LFS, and which can be derived from a postal code by someone with the knowledge of how to do so. Because this conversion is not obvious, the researchers tested matching with and without the OAC to see the results. Without OAC, they matched 20 records to 8 addresses, 2 of which were verified as true matches by the Office for National Statistics. With OAC, they matched 42 records to 27 addresses, 18 of which were confirmed to be true matches. In this case, more specific location information led to a greater number of individuals being re-identified.

Tudor, Spicer and Cornish [9], [10] conducted an intruder test on pre-publication census data in the UK to examine potential re-identification risk associated with the data release. The data they targeted was tabular in nature, consisting of 89 tables that were determined to be potentially high risk containing varying numbers of fields and more or less specificity in terms of location. The goal of the disclosure control techniques used for the 2011 Census was to create “sufficient uncertainty” as to the identity of any individual re-identified and targeted record swapping was chosen as the primary method of creating such uncertainty for tabular data. Not knowing which records have been swapped, an intruder in this case can never be sure if a re-identification results in a true disclosure. The authors recruited “intruders” from within the statistical agency to attempt to re-identify the data using only public information found on the web. They asked the volunteers to examine different re-identification scenarios, such as [9], [10]:

I. Can they identify themselves or their household?

II. Can they identify someone they know, either individually or within a group, and their characteristics?

III. Starting with public information, can they then identify someone, or a group of people, in a table (and learn more than the public information)?

IV. Starting with the census tables, can they identify a person or a group of people, and link this to some public information?

Eighteen intruders were recruited and there were more than 50 claims of identity and/or attribute disclosure from the group. The volunteers also noted the level of confidence they had in the correctness of their claims (from "Not at all confident" to "Very confident"). Researchers found that the volunteers claimed to identify only people about whom they had personal knowledge (save for 1 claim), and the claims were most accurate when they were about a family member or someone living in the same household as the intruder. In terms of confidence, the claims that testers had greater confidence in were more likely to be correct; however, there were more correct claims found at the "Reasonably confident" level than at the "Very confident" level [9], [10]. In the end, the majority of the claims were found to be incorrect, leading to the conclusion that "it is very difficult to re-identify respondents correctly in the 2011 UK Census and moreover, it is virtually impossible in this case to identify anyone correctly without any personal knowledge about them." [9], [10]

The UK Department of Energy and Climate Change underwent intruder testing prior to the release of the National Energy Efficiency Data (NEED) in 2014 [11]. The anonymized NEED data release consisted of 2 datasets: a public use file (PUF) of 50,000 records and an end user license file which includes 4 million records. Government analysts from the department's IT sector were recruited to act as motivated intruders, using publically available information in conjunction with their own knowledge to attempt to re-identify records in NEED. In almost every case, energy performance certificate (EPC) information was used to try to identify households as EPC data is both publically available and includes dates. As a result, the EPC variables were determined to be high risk and were aggregated or removed in the final data release. Further intruder testing of the PUF was conducted by post-graduate students in Electronics and Computer Science. The students were unable to correctly identify any household in the PUF dataset.

Ramachandran, et al. conducted a case study looking at the re-identification of sensitive data [12]. They examined the success of efforts to re-identify a large dataset by matching with a publically available dataset purchased from wholesale data sellers. The de-identified dataset examined contained over 2 million people. The public dataset they purchased for re-identification of the de-identified data contained demographic data for 700,000 people. The variables contained in the public dataset included name, date of birth, address, ethnicity, sex, and income. Using an algorithm to match the two datasets, researchers found that the probability of a successful match was less than 0.005%. They concluded that there is little risk posed by this type of large scale data matching attack.

The Heritage Health Prize dataset was made available for individuals to participate in a data analysis competition with an ultimate $3m cash prize [13]. This longitudinal claims dataset covered 113 thousand patients over a three-year period, accounting for re-admissions. Before the dataset was made available for the competition the sponsor commissioned a re-identification attack [14]. The attack explored multiple avenues to re-identify patients with different characteristics but did not re-identify any data subjects in the competition dataset.

These studies show that although large scale re-identification attacks are not likely to be very successful, more targeted attacks can be successful in re-identifying a small number of individuals. Also, the more background information or "response knowledge" an intruder has about an individual, the greater the risk that the individual could be re-identified.

# References

[1] Latanya Sweeney, Akua Abu, and Julia Winn, “Identifying Participants in the Personal Genome Project by Name,” Harvard University. Data Privacy Lab, 1021–1, Apr. 2013.

[2] K. El Emam, E. Jonker, L. Arbuckle, and B. Malin, “A Systematic Review of Re-identification Attacks on Health Data,” *PLoS ONE*, vol. 6, no. 12, 2011.

[3] M. J. Elliot and K. Purdam, “The Evaluation of Risk from Identification Attempts,” Manchester, UK, 5-D3, Sep. 2003.

[4] P. Kwok, M. Davern, E. Hair, and D. Lafky, “Harder Than You Think: A Case Study of Re-Identification Risk of HIPAA-Compliant Records,” in *JSM Proceedings*, Miami Beach, Florida, 2011.

[5] D. Lafky, “The Safe Harbor Method of De-Identification: An Empirical Test,” presented at the Fourth National HIPAA Summit West, San Francisco, CA, Oct-2009.

[6] Mark Elliot, “Using Targeted Perturbation of Microdata to Protect Against Intelligent Linkage,” in *Proceedings of UNECE Work Session on Statistical Confidentiality*, Manchester, United Kingdom, 2007.

[7] M. Elliot, “Report on the Disclosure Risk Analysis of the Supporting People Datasets,” Administrative Data Liaison Service, Mar. 2011.

[8] M. Elliot, E. Mackey, S. O’Shea, C. Tudor, and K. Spicer, “End User Licence to Open Government Data? A Simulated Penetration Attack on Two Social Survey Datasets,” *Journal of Official Statistics*, vol. 32, no. 2, pp. 329–348, 2016.

[9] C. Tudor, G. Cornish, and K. Spicer, “Intruder Testing on the 2011 UK Census: Providing Practical Evidence for Disclosure Protection,” *Journal of Privacy and Confidentiality*, vol. 5, no. 2, pp. 111–132, Aug. 2013.

[10] K. Spicer, C. Tudor, and G. Cornish, “Intruder Testing: Demonstrating practical evidence of disclosure protection in 2011 UK Census,” presented at the UNECE Conference of European Statisticians, Ottawa, ON, 2013.

[11] M. Gregory, “DECC’s National Energy Efficiency Data-Framework – Anonymised dataset,” Sep-2014.

[12] A. Ramachandran, L. Singh, E. Porter, and F. Nagle, “Exploring Re-Identification Risks in Public Domains,” presented at the 2012 Tenth Annual International Conference on Privacy, Security and Trust, 2012, pp. 35–42.

[13] K. El Emam *et al.*, “De-identification Methods for Open Health Data: The Case of the Heritage Health Prize Claims Dataset,” *Journal of Medical Internet Research*, vol. 14, no. 1, p. e33, Feb. 2012.

[14] A. Narayanan, “An Adversarial Analysis of the Reidentifiability of the Heritage Health Prize Dataset,” May 2011.
