# Supplementary material for: Evaluating the re-identification risk of a clinical study report anonymized under EMA Policy 0070 and Health Canada Regulations
Source: Trials. 2020 Feb 18;21:200. doi: 10.1186/s13063-020-4120-y (PMC7029478; doi:10.1186/s13063-020-4120-y)
Supplement: Supplementary file 2 — Additional file 2. Attack methods used. [file 13063_2020_4120_MOESM2_ESM.pdf]

Additional file 2: Attack Methods Used

The following sections will detail the goal, external datasets used, and outline of the attacks for each of these approaches.

B.1 Approach 1: Clinical Reports

One of the first avenues of attack consisted of matching external data for reported adverse events with the enriched data extracted from the clinical reports, launching a sample-to-population attack against other adverse event databases.

B.1.1 External sources of data examined

For this approach, the external sources consisted of two public databases maintained by the FDA in the US of adverse events reported. In particular these resources are the FAERS and MAUDE. For the EU, we looked at the EMA's EudraCT (European Clinical Trials Database) and the public EPAR (European Public Assessment Report) documents.

B.1.1.1 FAERS Database[1]

As defined by the FDA: "The FDA Adverse Event Reporting System (FAERS) is a database that contains adverse event reports, medication error reports and product quality complaints resulting in adverse events that were submitted to FDA". FAERS can be accessed and queried through a public online Qlik interface among which several searches and drilldowns can be performed to obtain a list of relevant case numbers.

However, it should be noted that the probability of re-identifying subjects using this specific method is quite low given that FAERS data contains Adverse Events for already approved drugs (i.e., post-marketing surveillance data rather than data from the actual trial); since it is unlikely that a subject suffered a similar drug-related adverse event that was recorded in FAERS.

B.1.1.2 MAUDE Database[2]

As defined by the FDA: "MAUDE data represents reports of adverse events involving medical devices. [...] The searchable database data contains the last 10 year's data". Given that all the subjects were diabetics, some of these patients should have insulin devices that may have malfunctioned, creating an adverse reaction that may have been recorded in the anonymized report. By searching through the "other drugs" that the patient with the malfunction was taking, we would be able to identify these potential cases.

[1] Link to FAERS: https://www.fda.gov/Drugs/GuidanceComplianceRegulatoryInformation/Surveillance/AdverseDrugEffects/ (last accessed March 2018)
[2] Link to MAUDE: https://www.fda.gov/medicaldevices/deviceregulationandguidance/postmarketrequirements/reportingadverseevents/ucm127891.htm (last accessed March 2018)

MAUDE data is available for download by year. Additionally, the FDA's MAUDE FAQ website states the availability of an online tool, but all the attempts we made to use it led us to the same FDA Internal Application Error page. For this reason, only the downloaded data was used to perform the attack.

B.1.1.3 FDA Public Data

For this approach, in addition to the sources discussed above, we examined other potential sources of information available around the drug Nevanac through the FDA site, and performed a review of the public facing documents related to that medication through the Drug Search. We searched for Nevanac and Nepafenac and reviewed all of the documents related to the drug that were returned from the Drug Search. We did not find anything that we could use to assist us with our attack.

B.1.1.4 EMA and EPAR Public Data

For this approach, the external sources consisted of public information available around the drug Nevanac, and a review of the public facing documents related to that medication. For the EMA site, we joined the EudraCT community, created a login and searched for relevant information and documents related to the drug and trial. We did not find anything that we could use to assist us with our attack.

Additionally, within the scope of the EMA, we obtained the public EPAR documents available for Nevanac. We reviewed the relevant documentation within that time frame, and were unable to find any information that we could use or leverage to help us with our re-identification attack.

B.1.2 Outline of the sample to population and population to sample attacks

As outlined above, we extracted the target data from the report and aggregated all the data available for each case with adverse events. This allowed us to put together a consistent dataset to refer to when trying a sample-to-population attack to the datasets mentioned in the previous subsection.

We looked through this sample one by one and attempted to find possible matches on both FAERS and MAUDE by similar type of effect description, gender and fuzzy age (since the age might have been masked). For each case, we compared the symptoms of FAERS and MAUDE to the effects and clinical reports from the enriched dataset extracted from the report, looking for matches between the two.

For each possible match we performed further analysis by comparing past medical history and other events described so as to better ascertain the probability of a match.

After analyzing all possible matches, we obtained six different cases that matched to the FAERS database with low probability of a match (given the fact that FAERS records approved drugs, as mentioned in the initial description of FAERS). In order to obtain more information about these cases we submitted a FOIA request to the FDA asking for the specific adverse event reports of each of these cases. Details and results obtained from the FDA FOIA request are reported in the section below, Approach 2: FDA and EMA (public and FOIA).

B.2 Approach 2: FDA and EMA (public and FOIA)

In this approach, we attempted to request information about the trial from agencies in the US and EU: the FDA and the EMA. Both FDA and EMA FOIA requests have potentially long response times that put them outside of the scope of what we are able to request within the time constraints of our investigation (for

example, see [1]). From conversations with the FDA, the FOIA request that we would most likely be able to fulfill in the timeframe of the investigation was limited to a small number of specific case records.

Following from the investigations described above in the previous sections, we determined six cases of potential matches for which to file FOIA requests. We filed FOIA requests for case records for each of the 6 case ids obtained from the FAERS database that matched the data obtained in the CSR, as detailed in Approach 1: Clinical Reports. Responses to our FOIA requests for specific matches one month after the request was made. The responses to the FOIA request were split between two types of documents: three of the six cases were case report information and the remaining three were scanned images of the individual safety reports of the specific adverse event.

B.2.1 Case Report Information

B.2.1.1 Contents

The case report information contained dates of the event, indications, drug information (treatment drug, dosage, route) and a small narrative. The event/problem narratives were very similar to the individual clinical report excerpts on the adverse effects section of the CSR, with some of the dates redacted. No location information or partially identifiable information was included.

B.2.1.2 Findings

The non-redacted dates did not provide additional information we could use to re-identify subjects, as they either confirmed or clarified information that we had already obtained from the CSR.

The report provided additional value to our process by confirming that the dates we had in the reports were the true dates (for those that were not redacted), age and gender (from the FAERS database), as opposed to the possibly masked or perturbed dates, age and gender from the anonymized CSR. This allowed us to slightly enhance our dataset.

In conclusion, the Case Report Information obtained through the FOIA requests did not provide us with any additional information to use and this information did not enable us, as attackers, to (fully or partially) de-identify any patients. To be able to use this information to (fully or partially) re-identify any patients, we would require additional external resources to match to the confirmed dates, age and genders.

B.2.2 Individual Safety Reports

B.2.2.1 Contents

The scanned individual safety reports contained classification, dates and descriptions of the adverse event; patient age; suspect product/device information; and concomitant medication. Additionally, the patient identifier, date of birth, medical personnel names, locations, and any phone numbers (of both the medical facilities or patient) are completely redacted.

B.2.2.2 Findings

As in the case report information described above, the only additional value obtained from these documents is the added certainty to some of the dates and the age and gender of the patient; but without any additional sources of information, based on solely these facts an attacker would not be able to

partially or fully re-identify the subjects of the trials.

Similarly, our conclusions from the review of the Individual Safety Reports from the FOIA responses did not provide us with any additional information to use and this information did not enable us, as attackers, to (fully or partially) re-identify any patients.

B.3 Approach 3: Death Records

The adverse effects reported five death events among the subjects of the clinical study. We attempted to re-identify these subjects by locating public death records and match them to the cause of death and approximately to the reported date of death range.

B.3.1 External sources of data examined

We found death records to be dispersed and decentralized, consisting of public records maintained at a county level, while some states have more centralized services but available only for mortuary professionals. Additionally, some paid subscription services exist for locating graves of family or determining possible ancestors are available via services such as ancestry.com. We confirmed with a mortuary operator that there was no public access to the information that they had specialized access to. Consequently, we used one of the most popular services (ancestry.com) to perform a sample-to-population attack.

B.3.2 Outline of the sample to population and population to sample attacks

The sample-to-population attack was performed by conducting searches through ancestry.com's site. However, given that we did not have names, we had to operate under the assumption that the age, gender and date of death were reasonably accurate (we tried performing some fuzzy matches, and looked into cases that were somewhat similar but did not match perfectly with all the parameters).

Searches were performed by these three parameters for each of the five death cases recorded. Searches were restricted to the entire US, since the probability of obtaining death records data of other countries was much lower. Although for other approaches we limited further the location to smaller areas (states or metropolitan areas) where clinical study had been conducted. Given the low number of death cases, we were able to proceed and comb through all the results without further restricting the location.

An additional challenge we encountered was the multiplicity of some death records on their site, probably due to the consolidation of different systems that might have had some overlapping data. However, we believe that had no impact on the results we obtained since no duplicates were found for the potential matches.

B.4 Approach 4: Hospital-specific Discharge Records

In order to be able to match to hospital discharge records, we set to find hospital locations on which the clinical trial was likely to have been performed. Once we obtained these locations, we would try to obtain discharge records and try matching them with the target dataset (population-to-sample attack).

B.4.1 Outline of the sample to population and population to sample attacks

We used public sources from clinicaltrials.gov and research databases such as Google scholar to find

locations, hospitals and institutions involved with the study to determine locations to assist with our attack strategy. Using Google Scholar, we were able to locate the published papers that referred to this specific study, and potential similar studies. The researchers publishing the paper list financial ties and the research units they belong to. Looking at clinicaltrials.gov revealed similar studies to the one cited and by comparing the two we were able to determine with a reasonable level of certainty possible hospitals or metropolitan areas in which the study could have been conducted. Optimizing within the constraints and limitations of the study, we focused the population-to-sample attack to the areas we had the most confidence in being part of the study: The Fort Worth metropolitan area and Houston medical centers.

However, after locating hospitals in these areas, in order to obtain data from these specific hospitals formal requests have to be submitted and in most cases approved by the Texas Health Resources IRB[3] and be de-identified. Access to de-identified hospital discharge records would not have added to our ability to re-identify participants. This avenue of attack was then unsuccessful and did not produce any potential matches.

B.5 Approach 5: Using Subject Recruitment Methods

This approach considered the ability to recruit subjects for medical studies as a means of finding subjects who may overlap with the subjects in this report. The set of inclusion criteria is relatively restrictive (especially due to the fact that the subjects had retinopathy surgery during a specific window of time). One possible attack vector we explored was obtaining subjects with similar characteristics, with the added requirement that they had participated in a past study within the time frame of the study report. To investigate this approach, we explored the possibilities of recruiting the same participants from professional medical subject recruiters and what kinds of information we could get on the potential subjects. Essentially, we explored the possibility of attempting to re-recruit individuals and from there try to identify any possible overlap and matches to the original study.

B.5.1 External sources of data examined

We were able to interview several people that work in or closely related to medical recruiting firms, the insights of which are detailed in the next subsection. No further resources were used.

B.5.2 Outline of the sample to population and population to sample attacks

We looked into online recruiting methodologies and existing recruiting companies as an option. For example, a medical recruiter allows a researcher to specify very precise criteria for selecting subjects including condition, demographics, etc. By specifying the adapted criteria that were in our study, we would be able to recruit subjects that may contain some overlap with the target population; after which we could match like attributes to infer an actual identity.

We found that there were two major obstacles that prevented us from moving forward with this approach. The first was payment and scale, and the second was the limitations on the data we would potentially acquire as a result of this recruitment. In order for us to obtain a sufficient amount of subjects so as to

[3] As specified in the research data request information webpage of Texas Health. Link: https://www.texashealth.org/research/research-development/research-data-request (last accessed on March 2018)

have a reasonable probability of overlap we would have had to recruit a sizable amount of subjects and we determined that the expense was not justifiable with the expected probability of finding potential matches.

Furthermore, this information is often de-identified with efforts made to remove personal information. This would the create the need to at first re-identify that data set in order to then apply that against the target data set, making for an unreliable ground truth.

B.6 Approach 6: Social Media Attacks

Although subjects of clinical studies should not publicly post information about their study, the use of social media is so pervasive that some people may, unknowingly, divulge partial identifiers that would make it possible to identify them as subjects of the study. We explored multiple different social media platforms and performed both population-to-sample and sample-to-population attacks as detailed in the following subsections.

B.6.1 External sources of data examined

We performed searches across various social networking sites, looking for public groups, members on Facebook, Reddit, Twitter and online help groups.

B.6.2 Outline of the sample to population and population to sample attacks

We performed two different types of searches: keyword-searches among personal posts or comments, and searches among help groups (both of posts and members).

For the population-to-sample social media attacks, we first compiled a list of keywords (see *Appendix D – Search Keywords* for list of keywords) that included the name of the drugs (real name and potential alias used during the study), the surgical procedure, common reactions and effects and other terms related to the clinical study performed. These keywords were then searched among Facebook posts, Twitter posts and general internet blog posts in order to identify users that could be potential matches, as detailed below.

We also ran sample-to-population (or hybrid) attacks in which we used the keywords to look for help groups in Facebook (pages and groups), Reddit discussion groups and other online health groups. We then proceeded to look among the members for people that fit and combine both traits: diabetes and cataract surgery within a specific time period. For those, we tried to match them to possible characteristics of the people that we had in our anonymized report through adverse events or any other information that may help identify them as participants of this particular study.

A brief description of each attack for both methodologies follows.

B.6.2.1 Facebook Keyword-Search of Posts

Facebook's developer API does not allow for general searches of posts, however any user can search for keywords among the entire corpus of public posts made by all Facebook users. Using such functionality we ran keyword searches for the specific years of the clinical trial (March 2013 to May 2015) and identified several posts that may have been posted by potential subjects.

One major challenge with this search tool is that it is not possible to combine multiple keywords or refine the search results (aside from restricting the post date). Therefore the approach we found more productive and efficient was to first identify cataract surgery patients with fully public posts, and then try to identify if they were diabetic among their personal posts, pictures, and personal information posted.

B.6.2.2 Twitter Keyword-Search of posts

Similar to the Facebook approach, we were able to perform keyword searches of public tweets. This time we were able to create a small tool to sift through the results of a specific search and perform further breakdowns or additional filters by other keywords (see *Appendix – Searching Keywords* for a list of keywords).

The challenge in this case was the low amount of real user posts, as opposed to ads or professional posts among the hits. Additionally, the short nature of the text and relatively limited amount of information relayed by Twitter user's on their profile rendered all our efforts fruitless; not being able to identify a single match with a reasonable probability of being a subject of the target dataset.

B.6.2.3 Facebook Keyword-Search of help groups and pages

Facebook has multiple groups or pages in which users bond and share similar experiences; health groups are among one of the typical uses of these sub-communities. The Facebook site allows users to search for groups by keyword, not only among the title or description of these groups but also the posts in those groups that have been set to public.

We ran searches using the keywords from the *Appendix – Searching Keywords* and were able to identify a few groups with public settings, or at least public listing of their members. For those groups with public member groups, we could look at the personal posts of those members who had public profiles.

No public groups were identified for diabetics with eye problems subjects in the USA during the time of the clinical trial (the groups identified were created post-2014). Among the multiple groups found of either diabetes or cataract surgery no indication was found for possible members suffering the other condition.

Several non-public groups for diabetics with eye problems were identified. For these, we combed the member list and went through each of the members that had a public profile. We tried to locate specific mentions of when the surgery was performed or clinical visits (to try to match to our clinical study period). However, Facebook's limited functionality for narrowing down results forced us to perform most of these searches manually through the subject's profiles. No positive results were found.

B.6.2.4 Reddit and Blogs Keyword-Search of help groups and pages

Reddit has a diabetes forum that includes both type 1 and 2 diabetes. Within this discussion group we searched for any mentions of cataracts or other related eye problems similar to those seen on the report.

The few cases we found with explicit mentions of cataract surgery did not fall in the time of the study. Only one case mentioned a surgery date that was within the clinical study, however in that case the commenter was posting on behalf of her father. The limited public information necessary to post on Reddit complicated our efforts to obtain more information. We were only able to use typical post times and other casual posts to determine her location to most probably be North America. No further information was obtained from this case, and no additional potential matches were identified using Reddit.

B.7 Approach 7: Voter Registration Records

Previous attacks have outlined the possibility of using Voter Registration Records as a means of re-identifying personal health records8. We looked into the possibility of using Voter Registration Records to enhance our dataset to assist with re-identification, but ultimately concluded we would be unable to use them and they would not help us. We found that there was not enough information from our data subjects in the report to reliably match them up against potential matches from voter registration records, for example state/location information and DOB or other identifiers we would need to potentially link identities. Consequently, we were not able to use Voter Registration Records.

B.8 Other Approaches: Enhancing dataset and study details

Additionally, other sources were used to try to enhance our dataset that might be helpful when trying to identify the subjects with the sources above mentioned.

The website clinicaltrials.gov is an updated registry of all clinical trials in the US, both completed and running. We were able to identify the study and combed through the supplementary information and attached documents. However, the information did not contain any subject-level or aggregate level details beyond those found in the research papers: possible locations of the study based on the research centers involved and disclosed financial sponsors; most of the documents focused on the chemical aspects of the drugs and the protocols followed during the approval of the drug.

References

[1] P. Doshi and T. Jefferson, "Open data 5 years on: a case series of 12 freedom of information requests for regulatory data to the European Medicines Agency," *Trials*, vol. 17, p. 78, 2016.
