# Supplementary material for: Evaluating the re-identification risk of a clinical study report anonymized under EMA Policy 0070 and Health Canada Regulations
Source: Trials. 2020 Feb 18;21:200. doi: 10.1186/s13063-020-4120-y (PMC7029478; doi:10.1186/s13063-020-4120-y)

Additional file 3: Effort Diagram per Approach

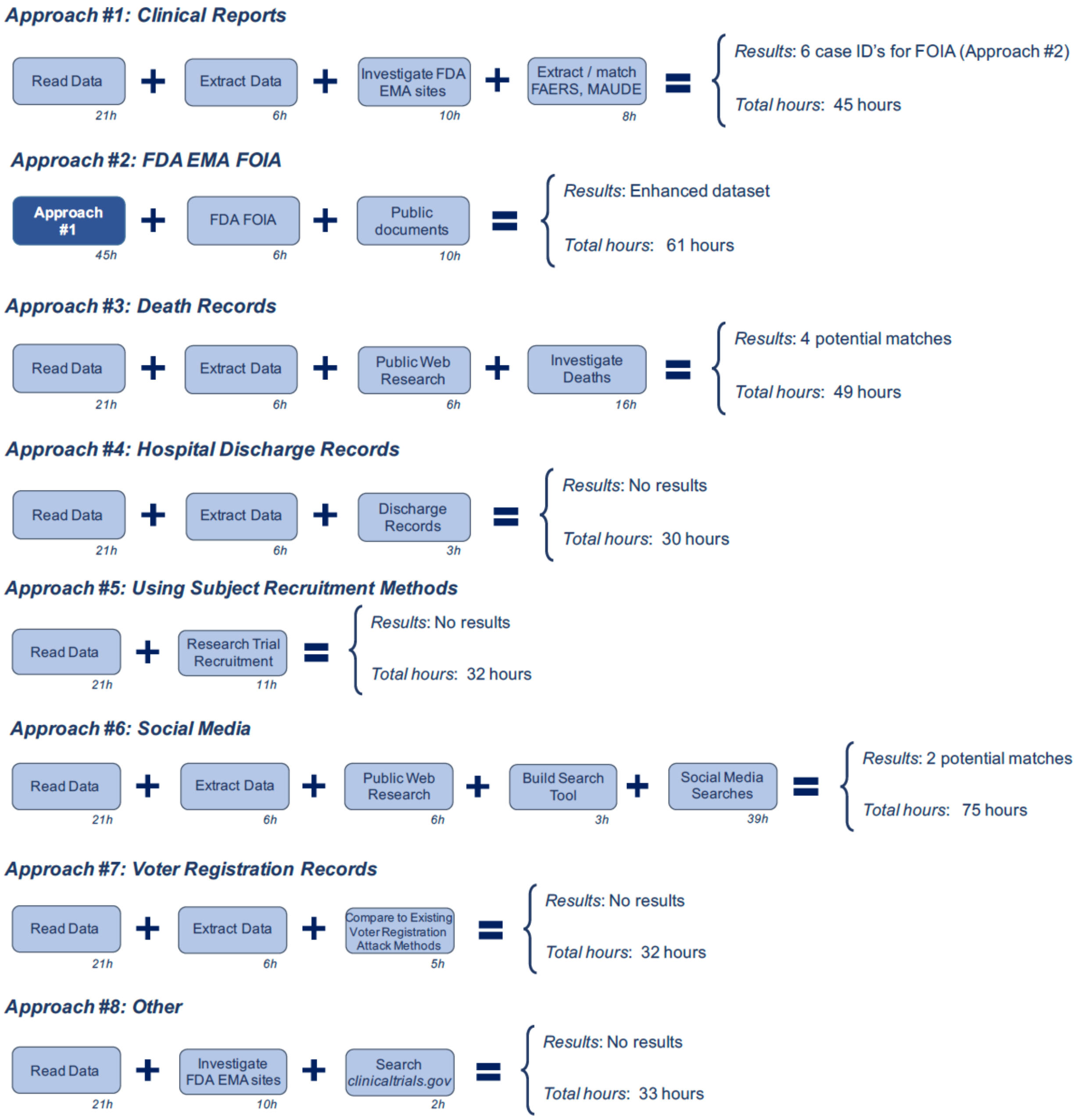

Supplement: Supplementary file 3 — Additional file 3. Effort diagram per approach. [file 13063_2020_4120_MOESM3_ESM.pdf]
