# Supplementary material for: Evaluating the re-identification risk of a clinical study report anonymized under EMA Policy 0070 and Health Canada Regulations
Source: Trials. 2020 Feb 18;21:200. doi: 10.1186/s13063-020-4120-y (PMC7029478; doi:10.1186/s13063-020-4120-y)
Supplement: Supplementary file 4 — Additional file 4. Search keywords. [file 13063_2020_4120_MOESM4_ESM.pdf]

Additional file 4: Search Keywords

- diabetes
- insulin
- cataract
- surgery
- NPDR (nonproliferative diabetic retinopathy)
- retinopathy
- eyesight failing
- cataract surgery
- study
- clinical trial
- test
- macular edema
- medication
- Nepafenac (although during the study the patients may not have known the name)
- Nevanac (although during the study the patients may not have known the name)
